# Economic Burden of Inflammatory Bowel Disease in Shiraz, Iran

**DOI:** 10.34172/aim.2023.04

**Published:** 2023-01-01

**Authors:** Leila Rahmati, Alireza Mooghali, Seyed Mohammad Hosein Kamani, Fatemeh Zare, Hassan Askari, Ali Reza Safarpour

**Affiliations:** ^1^Gastroenterohepatology Research Center, Shiraz University of Medical Sciences, Shiraz, Iran, Department of Management, Payame Noor University, Tehran, Iran; ^2^Department of Management, Payame Noor University, Tehran, Iran; ^3^Department of Research Deputy, School of Medicine, Shiraz University of Medical Sciences, Shiraz, Iran

**Keywords:** Direct costs, Economic burden, Indirect costs, Inflammatory bowel diseases

## Abstract

**Background:**

The epidemiological burden of chronic diseases and their risk factors is increasing all over the world, especially in developing and low-income countries. Inflammatory bowel disease (IBD) is one of the chronic diseases which has imposed a great financial burden on individuals and the society.

**Objectives:**

The current study aimed at estimating the economic burden of IBD among 90 patients with IBD who referred to Namazi hospital and Motahari clinic of Shiraz in 2019. The costs to patients were monitored for a year to detect their expenses.

**Methods:**

This study is descriptive cross-sectional and from a social perspective. The cost-of-illness method, based on the human capital theory, has been used. Both direct and indirect costs have been estimated using a prevalence approach and bottom-up method. Hospital costs were extracted from patients’ records and the accounting system of Namazi Hospital. Outpatient expenses were obtained according to the number of outpatient visits and the average cost of visit were obtained by interviewing patients. Socio-economic status, medical expenses and number of days absent from work were determined using a valid and reliable questionnaire. Assessment of the cost of hospital care was made on the basis of the average daily. Non-medical direct costs such as transportation and residence, etc. were also calculated.

**Results:**

The total annual economic costs of IBD per patient were estimated at 1229.74 USD. Finally, increased use of health care as well as lost productivity leads to increased disease costs.

**Conclusion:**

IBD imposes a substantial economic burden on patients, families and the society. Establishing a correct diagnosis early, management of IBD worsening, and appropriate treatment can reduce the costs of treatment and lost production to some extent. Therefore, policymakers should take this into consideration and according to available health resources, provide services and facilities for the prevention and treatment of the disease.

## Introduction

 Inflammatory bowel disease (IBD) is a chronic, lifelong condition characterized by inflammation of the gastrointestinal tract with recurrent episodes of relapse and remission. It includes Crohn’s disease and ulcerative colitis (UC).^1,2^ UC is an inflammation of the large intestine that starts in the anus and can spread to other parts of the bowel and has symptoms such as diarrhea and rectal bleeding, while Crohn’s disease can affect any part of ​​the gastrointestinal tract and is accompanied by symptoms such as diarrhea, abdominal pain, and weight loss. Crohn’s disease and UC are controllable, but incurable diseases with extra-gastrointestinal side effects such as skin, eyes, and joints involvement.^3^ IBD can affect anyone at any age but usually begins between the ages of 15 to 30 years. UC affects both sexes equally.^4,5^ The goal of treating IBD is to control the acute phase of the disease and eliminate or reduce the symptoms and then, to maintain the remission phase using medications, dietary changes, and surgery.^6^ The annual incidence of Crohn’s disease in the US is estimated at 6 to 8 cases per 100 000 people and its prevalence is estimated at 200 to 300 cases per 100 000. The incidence of IBD varies in different geographical regions. The highest incidence of UC and Crohn’s disease has been reported in Europe, the UK, Italy, Canada, and North America.^7-9^ In Iran, the epidemiological characteristics of IBD and its exact prevalence have not been determined yet, and studies have only been conducted on a population of patients, mostly because of the lack of a national disease registration system and the presumption of Crohn’s disease rarity in the country.^10^ Reports indicate that the prevalence of IBD has increased from 4.69 per 100 000 people in 1990 to 40.67 per 100 000 in 2012.^11^ The disease also causes disability and morbidity, especially in young adults, and imposes a great social and economic burden by affecting the daily activities of patients who had the potential to grow, study, and work.^12^ Despite having the lowest prevalence among gastrointestinal diseases, IBD ranks first among the five most costly.^13^ Despite the impact of IBD on the quality of life and efficiency of people and the great financial burden it imposes on the national health system, no studies have yet examined the costs of this disease in Iran. The present study is the first attempt to estimate the economic burden of IBD in Shiraz, Iran and determine its direct and indirect costs so as to recognize the costs it imposes on the health system and to help in optimal resource allocation and effective cost management in this regard.

## Patients and Methods

 This study used the cost-of-illness method,^14^ utilizing the prevalence-based approach and the bottom-up method, to calculate the economic burden of IBD, and the costs were examined from a social perspective. The study population consisted of all IBD patients who visited Motahari Clinic and were hospitalized at Namazi Hospital in Shiraz in 2019. Using census, the number of these people was found to be 125; an attempt was made to interview all of them but for various reasons, including lack of access and inaccurate data on the diagnosis and death of the subjects, 35 people were excluded from the study, and the information of 90 subjects was examined. Table 1 presents the primary information of the included patients. Questionnaires and interviews were data collection instruments. Accordingly, patients’ files were reviewed by referring to Motahari Clinic in Shiraz, and patient history and information on costs were gathered by interviewing the patients. The questionnaire was a standard cost-of-illness questionnaire prepared based on a questionnaire used in a previous study^15^ and in line with the present study objectives. This standard questionnaire included data related to the use of inpatient and outpatient services; patient characteristics such as age, gender, educational level, employment status, and insurance coverage; and direct treatment costs including the number of visits to physicians’ office, medications used, length of hospital stay, surgery, and other therapeutic measures. Besides, the number of days the patients and those looking after them missed work and costs of other services used outside the health system were considered.

**Table 1:** Demographic and Personal Data of All IBD Patients Who Visited Motahari Clinic and Were Hospitalized at Namazi Hospital in Shiraz, Iran

**Demographic Characteristics **	**UC ** **No. (%)**	**CD ** **No. (%)**	**IBD ** **No. (%)**
Age			
Under 20	12 (20.4)	19 (61.3)	31 (34.5)
21-40	16 (27.2)	8 (25.8)	24 (26.6)
41-70	27 (45.8)	3 (9.7)	30 (33.3)
70 and over	4 (6.8)	1 (3.2)	5 (5.6)
Gender			
Male	25 (42.4)	17 (54.8)	42 (46.7)
Female	34 (57.6)	14 (45.2)	48 (53.3)
Education			
High school or below	38 (64.4)	24 (77.5)	62 (69)
College graduate	11 (18.7)	6 (19.4)	17 (18.9)
Bachelor's degree or higher	10 (17)	1 (12.9)	11 (12.2)
Geographic location			
Urban	21 (35.6)	13 (41.9)	34 (37.8)
Rural	38 (64.4)	18 (58.1)	56 (62.2)
Job			
Civil servant	5 (8.5)	0	5 (5.6)
Private sector employee	3 (5.1)	2 (6.5)	5 (5.6)
Self-employed	6 (10.2)	4 (12.9)	10 (11.1)
Retired	7 (11.9)	0	7 (7.8)
Unemployed	31 (52.5)	12 (38.7)	43 (47.8)
Student	6 (10.2)	13 (41.9)	19 (22.2)
Insurance status			
Yes	59 (100)	28 (90.3)	87 (96.6)
No	0	3 (9.7)	3 (3.4)

UC, Ulcerative colitis; CD, Crohn’s Disease; IBD, Inflammatory bowel disease.

###  Economic Burden Calculation 

 The total economic burden of diseases consists of two parts: (*i*) the direct economic burden, which itself consists of direct medical costs and direct non-medical costs, and (*ii*) indirect costs. Direct medical costs were measured taking into account the average outpatient and inpatient costs, and costs of medication, diagnostic care, physician visits, and other therapeutic measures for subjects in the last quarter of 2019. The cost of physician’s office visits for these patients was calculated taking into account the frequency of patient’s visit to the physician’s office and the cost per visit based on various physicians’ tariff rates in 2019. Diagnostic services, including blood and stool tests, endoscopy, colonoscopy, ultrasound, etc. used by the IBD patient in the last quarter of 2019 were also included in the questionnaire, and the total diagnostic cost was determined by calculating the cost of each test. The cost of medication was calculated by determining the type and amount of medications used by patients and the cost of each medication, using the information available from pharmacies. The expenses of patients who were hospitalized during this period were determined by referring to the accounting department of Namazi Hospital and receiving the bills of these patients. Non-medical direct costs were calculated using the standard questionnaire and through interviews with IBD patients. Since most of the referred patients lived outside of the city of Shiraz, items such as travel expenses of the patients and their family to medical centers and accommodation costs were considered as important components of direct non-medical expenses. The human capital method was used to calculate indirect costs.^16^ This approach measures health in terms of improvement in productivity and income (especially by reducing absence from work and increasing life expectancy). Thus, the indirect costs of illness are calculated based on the number of days of absence from work due to disability or because patients and those looking after them were busy seeking and pursuing treatment. The number of disability days was multiplied by the average daily wage in the year under study (according to the Statistics Center of Iran) to determine the indirect cost to employed patients. Since unemployed people with IBD cannot carry out their daily activities, a wage rate equal to the official minimum daily wage in 2019 was considered for these people. The cost of disability for those looking after the patient was considered as equal to the minimum daily wage, as for the unemployed. In order to obtain the annual total costs, the expenses obtained for three months were multiplied by 4. Data were presented in means and percentages, and all descriptive and inferential statistics were calculated using SPSS-23.

## Results

 Of 90 patients studied, 34.4% had Crohn’s disease and 65.6% UC; 42 were male and 48 were female. The mean age of the patients was 34.62 years, with a standard deviation of 23.35. The minimum age was 1 year and the maximum age was 94 years. IBD was most prevalent in the under-10-years age group. Among the patients, 11 (12.2%) had a bachelor’s or higher degree, 17 (18.9%) had a high school diploma or advanced diploma, 38 (42.3%) had an education below high school diploma, and 24 (26.7%) were illiterate. Also, 41.1% were single, and the rest were married. Among the employed patients, 8.9% had an average monthly income of 83.3–166.6 USD and 21.1% had an average monthly income of 166.6–250 USD. Also, 87 were covered by insurance, 3 were not insured, and 28 had supplementary insurance coverage. Of the subjects, 44.4% had Social Security insurance, 22.2% rural insurance, 8.9% Treatment Services insurance, and 6.7% Health insurance. Moreover, 52.3% were employed and the rest, unemployed. Of the patients, 72.2% were hospitalized for treatment with a mean length of stay of 13.43 days, while the minimum length of hospital stay was 1 day and the maximum length was 55 days.

 As Table 2 shows, the total direct medical costs of IBD in the study in 2019 was 868.39 USD on average. According to the table, among the components of direct medical costs, the highest percentage was related to hospitalization costs (54.63%) followed by medication costs (34.35%).

**Table 2:** Estimation of Direct Medical Costs of UC, CD, IBD by Types of Costs Per Patient in 2019 (USD)

**Cost Category **	**UC (Mean±SD)**	**CD (Mean±SD)**	**IBD (Mean±SD)**	**Percentage**
Medication costs (Min, Max)	288.7163 ± 490.1736 (0, 2149)	316.7156 ± 474.6728 (0, 1498)	298.3605 ± 482.3983 (0, 2149)	(34.35)
Diagnostic costs (Min, Max)	29.66801 ± 56.95142 (0, 250)	29.29834 ± 60.12851 (0, 250)	29.54075 ± 57.72733 (0, 250)	(3.40)
Physician's office visits (Min, Max)	31.40675 ± 17.92434 (0, 87)	32.80642 ± 20.93175 (0, 69.5)	31.88892 ± 18.90792 (0, 87)	(3.67)
Outpatient care costs (Min, Max)	27.74009 ± 190.4045 (0, 1450)	45.72576 ± 1220.663 (0, 395)	33.93517 ± 172.0354 (0, 1483)	(3.95)
Hospitalization costs (Min, Max)	528.4747 ± 810.2324 (0, 4317)	371.5808 ± 15.4343 (0, 1417)	474.4335 ± 701.1504 (0, 4317)	(54.63)
Total direct medical costs (Min, Max)	906.0059 ± 970.7342 (17.5, 4584.5)	796.8045 ± 729.3925 (27.5, 2961)	868.3922 ± 892.2738 (17.5, 4584.5)	100

UC, Ulcerative colitis; CD, Crohn’s Disease; IBD, Inflammatory bowel disease; SD, Standard deviation.

 The costs of inter-city and inside-city travel to medical centers for patients and those looking after them and their accommodation costs were calculated to determine the direct non-medical costs. These costs were calculated by multiplying the number of patients by the mean cost of transportation, accommodation, etc. As Table 3 shows, the direct non-medical costs of IBD per patient were 44.68 USD. The largest share of direct non-medical expenses was related to travel expenses (66.5%) because of the high frequencies of visits to physician’s office or hospitalization. Based on the responses received from the subjects, a high percentage of the patients’ caregivers stayed at relatives’ homes or in the hospital.

**Table 3:** Direct Non-medical Costs of UC, CD, IBD Patients Per Patient in 2019 (USD)

**Type of Costs**	**UC (Mean±SD)**	**CD (Mean±SD)**	**IBD (Mean±SD)**	**Percentage**
Transportation costs (Min, Max)	27.6 ± 49.81 (0, 250)	33.8 ± 51.2 (0, 240)	29.7 ± 50.1 (0, 250)	(66.5)
Accommodation costs (Min, Max)	9.06 ± 31.61 (0, 183)	27.4 ± 61.9 (0, 250)	15.4 ± 45 (0, 250)	(33.5)
Total direct non-medical costs (Min, Max)	36.661 ± 61.97 (0, 279)	59.96501 ± 103.9 (0, 457.5)	44.68792 ± 79 (0, 457.5)	100

UC, Ulcerative colitis; CD, Crohn’s Disease; IBD, Inflammatory bowel disease; SD, Standard deviation.

 As Table 4 shows, the total indirect costs of IBD were about 316.66 USD on average per patient in this study in 2019. The largest share of indirect costs was related to the indirect costs of the patient due to absence from work (55.29%).

**Table 4:** Indirect costs of UC, CD, IBD Patients and Their Accompaniers Per Patient in 2019 (USD)

**Type of Costs**	**UC (Mean±SD)**	**CD (Mean±SD)**	**IBD (Mean±SD)**	**Percentage**
Costs of patients’ missed opportunities (Min, Max)	182.5 ± 350 (0, 1800)	161 ± 377 (0, 1800)	175 ± 357 (0, 1800)	(55.29)
Cost of accompaniers’ missed opportunities (Min, Max)	107.5 ± 166.7 (0, 600)	206.4 ± 210.5 (0, 900)	141.5 ± 187.6 (0, 900)	(44.71)
Total indirect costs (Min, Max)	289.9718 ± 373.7 (0, 1800)	367.4731 ± 349 (0, 1800)	316.6667 ± 365.3 (0, 1800)	100

UC, Ulcerative colitis; CD, Crohn’s Disease; IBD, Inflammatory bowel disease; SD, Standard deviation.

**Figure 1:**
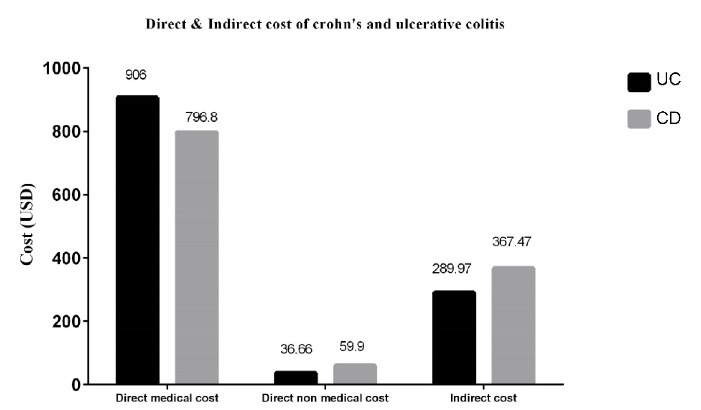
Schematic Presentation of Direct Medical Cost, Direct Non-medical Cost and Indirect Costs of Inflammatory Bowel Diseases. In terms of direct medical costs and indirect costs, ulcerative colitis is more costly. UC, ulcerative colitis; CD, Crohn’s disease.

 As Table 5 shows, the economic burden of IBD for the study population per patient in 2019 was estimated at an average of 1229.74 USD. In this study, direct medical costs accounted for the largest share (70.61%) of the total economic costs, and indirect costs due to reduced productivity of the patients constituted 25.75% of the total economic burden of IBD. Figure 1 shows that direct medical costs and indirect costs accounted for the largest share of costs in UC, and in general, direct medical costs and indirect costs created more financial burden for UC.

**Table 5:** Total Economic Costs of UC, CD, IBD Per Patient in 2019 (USD)

**Type of costs**	**UC (Mean±SD)**	**CD (Mean±SD)**	**IBD (Mean±SD)**	**Share of Each Cost of the Total Cost (%)**
Direct medical costs (Min, Max)	906.0059 ± 970.7342 (17, 4584)	796.8045 ± 729.3925 (27, 2961)	868.3922 ± 892.2738 (17, 4584)	70.61
Direct non-medical costs (Min, Max)	36.661 ± 61.97325 (0, 279)	59.96501 ± 103.8894 (0, 457)	44.68792 ± 79.15184 (0, 457)	3.63
Indirect costs (Min, Max)	289.9718 ± 373.7936 (0, 1800)	367.4731 ± 348.9226 (0, 1800)	316.6667 ± 365.3278 (0, 1800)	25.75
Total economic costs (Min, Max)	1232.639 ± 1406.501 (17, 6049)	1224.243 ± 1182.205 (87, 3718)	1229.747 ± 1336.754 (17, 6049)	100

UC, Ulcerative colitis; CD, Crohn’s Disease; IBD, Inflammatory bowel disease; SD, Standard deviation

## Discussion

 Chronic diseases are among the conditions with the most negative impact on the general health of people in the community. The protracted suffering from these diseases, the lengthy process of their treatment, their complications, and the fact that there is no appropriate and definitive treatment for most of them, have turned chronic diseases into a source of attrition in public health. As the incidence and prevalence of IBD are increasing worldwide, the financial burden it imposes on the health system and the economy as a whole will grow significantly.^17,18^ Even though the economic burden of IBD in Iran is low compared to developed countries, it is very high for Iranian households considering their average income. Awareness of the costs of these diseases and their management not only reduces the direct financial costs, but also contributes to the prosperity of the society by improving people’s quality of life and, consequently, their productivity. There have been several published studies on the economic burden of IBD worldwide, but no study has been published on IBD costs in Iran. Therefore, the present study attempted, for the first time in Shiraz, Iran, to estimate the direct and indirect costs associated with IBD in terms of the loss of productivity or absence from work due to the disease. Since the first economic study on IBD by Pinchbeck et al^19^ in 1988, more than 20 studies have been published on the subject; however, these studies lacked many essential details, such as the calculation of non-medical direct costs, while these costs are examined in the present study. Failure to take these costs into account in economic analyses leads to underestimating disease costs.^20^ In our study, many patients stated that they had to borrow from relatives, sell their assets, and get loans to pay for their treatment, and thus their treatment process severely affected household income. This issue was more pronounced in the case of special drugs or medical services provided by private centers not covered by insurance. According to the results, the direct medical costs for each patient with Crohn’s disease and UC in 2019 were estimated at 796.80 and 906 USD, respectively, and the direct non-medical costs were estimated at 59.96 and 36.66 USD, respectively. Also, the indirect costs of the disease per patient in 2019 for Crohn’s disease and UC were estimated at 367.47 and 289.97 USD, respectively. In this study, compared to Crohn’s disease, UC posed a greater economic burden due to the higher number of patients with UC. This finding is consistent with studies by Malekzadeh et al^11^ and Kamat et al.^20^ IBD imposed an economic burden of 1229.74 USD per patient, of which 70.61% (868.39 USD) was related to direct medical costs (including the costs of outpatient visits and hospitalization, diagnostic procedures, medication, and other services and treatment processes) and 25.75% (316.66 USD) was related to the cost of lost production due to disability. Also, the cost of hospitalization and medications were important factors in increasing the economic costs of the disease. The high percentage of hospitalization costs in the total direct medical expenses is due to the fact that patients must be hospitalized to receive injections of some types of drugs or undergo surgery for IBD. Lack of insurance coverage, high cost of medicines, and failure to adhere to treatment further increase the costs for IBD patients. The results of the studies conducted in the United States^21^ and Europe^22^ showed that hospitalization accounts for the largest part of the direct medical costs of IBD, which is consistent with the present study. Luces and Bodger^23^ examined the economic burden of IBD in the UK and showed that hospital costs accounted for almost half of all direct costs, and medication costs accounted for less than a quarter of the medical care costs. Their results are consistent with the findings of the present study. In a study by Ali Baig et al^24^ in 2017, the mean (direct and indirect) cost per patient over six months was 26,394 Rs (Hyderabadi rupees) and the costs imposed on patient caregivers due to the loss of working days accounted for 45% of the total indirect cost. This finding is consistent with the results of the present study. A study by the European Collaborative Studies Group showed that the average annual costs of IBD was 1871 EUR per patient in 2004. Hospitalization costs accounted for the largest share of the costs for both patients with UC (63%) and Crohn’s disease (45%). Their results are consistent with the present study to some degree.^25^

 The limitations of this study are its flaws in sample size due to chronicity of disease and participation refusal during follow-up. These limitations led to shortcomings in generalizability of our result. Another limitation of the study was that, due to the lack of access to the relevant data, it was not possible to estimate some costs, such as costs related to home care and unconventional and complementary therapies, and hidden costs such as those related to pain and depression. Despite these limitations, the present study has some strengths such as estimating various cost components, including the cost of transportation and lost production, and the cost of disability for patients and their families due to inpatient and outpatient care, which are usually ignored in other studies.

 In conclusions, specific standard of care for treatment of IBD that effectively controls disease activity, disease severity and early diagnosis before 2 years, can prevent bowel damage. This strategy reduces repeated hospital admissions and surgery, and subsequently reduces the costs of treatment. Therefore, health policy-makers and managers can plan and allocate resources based on the evidence and results presented in this study. Quantitative and qualitative improvement of the health insurance for IBD patients to help them deal with devastating costs of the disease, giving government subsidies to reduce the price of expensive medications, reforming the current payment system, and moving toward prospective payment systems are among the necessary measures to reduce the financial burden of IBD. Future studies are recommended to determine the cost-effectiveness of various IBD treatments, model factors that affect IBD costs, examine the impact of IBD costs on national economic growth, and explore effective IBD cost reduction strategies.
